# CD1d remodels the tumor-infiltrating myeloid populations controlling antitumor immunity

**DOI:** 10.1084/jem.20260216

**Published:** 2026-08-13

**Authors:** Lauren Evans, Maria Conde Poole, Cenk Celik, Harshita Mishra, Michael J. Pitcher, Anita Grigoriadis, Maria Secrier, Patricia Barral

**Affiliations:** 1 Department of Inflammation Biology, Centre for Inflammation Biology and Cancer Immunology, King’s College London, London, UK; 2 The Francis Crick Institute, London, UK; 3 Department of Genetics, Evolution and Environment, UCL Genetics Institute, University College London, London, UK; 4 Cancer Bioinformatics, Faculty of Life Sciences and Medicine, King’s College London, London, UK

## Abstract

Myeloid cells play crucial roles in cancer progression, influencing tumor growth, metastasis, and response to immunotherapy. The mechanisms shaping their diverse functions in the tumors remain poorly understood and may offer therapeutic opportunities. Here, we identify the lipid-presenting molecule CD1d as a regulator of tumor progression and myeloid heterogeneity in the tumor microenvironment. Using several mouse models of breast cancer, we demonstrate that genetic deletion or antibody-mediated targeting of CD1d leads to reduced tumor growth, altered immune infiltration, and improved efficacy of anti-PD-1 immunotherapy. Specifically, CD1d targeting reshapes the intratumoral myeloid compartment, enhancing proinflammatory programs and resulting in accumulation of inflammatory monocytes. The CD1d-dependent control of myeloid cell functional differentiation is cell-intrinsic and conserved in human and mouse. Through single-cell RNA sequencing, we define the transcriptional landscape associated with CD1d deficiency and derive a gene signature that correlates with clinical outcomes and response to immunotherapy in breast cancer patients. Thus, CD1d could provide a potential target to alter tumor-infiltrating myeloid populations and enhance immunotherapy responses.

## Teaser

Evans et al. identify CD1d as a key regulator of myeloid cell diversity in breast cancer. CD1d targeting reshapes tumor immune infiltrates, suppresses tumor growth, and enhances immunotherapy efficacy, suggesting an actionable pathway for therapeutic intervention.

## Introduction

Myeloid cells, including macrophages, dendritic cells (DCs), monocytes, and granulocytes, represent a major component of the tumor microenvironment (TME) in solid tumors, and they are critically involved in the regulation of tumor growth, vessel formation, metastasis, and response to treatment (Lopez-Yrigoyen et al., 2021). Beyond the classically activated/M1 and alternatively activated/M2 macrophage polarization model, which has been used to describe the activation state of macrophages *in vitro*, tumor-associated macrophages (TAMs) and monocytes are highly heterogeneous populations including diverse subsets and functional programs associated with inflammation, antigen presentation, phagocytosis, lipid metabolism, or immune regulation (Cheng et al., 2021). Within the TME, TAMs have been thought to be largely immunosuppressive and to aid tumor progression and metastasis by inactivating cytotoxic T cells, promoting angiogenesis, or supporting epithelial-to-mesenchymal transition (Cassetta et al., 2019; Lin et al., 2006). However, in recent years populations of TAMs and monocytes have been identified as promoters of antitumor responses and associated with better prognosis in patients with a variety of tumors (Acha-Sagredo et al., 2025; Clark et al., 2025; Elewaut et al., 2025; Nalio Ramos et al., 2022; van Elsas et al., 2024). TAMs are abundant in all solid tumors and readily adapt to changes in environmental cues, which renders them susceptible to therapeutic manipulation (Lopez-Yrigoyen et al., 2021; Mantovani et al., 2022). Nonetheless, direct targeting of TAMs for treatment has so far failed to have a major therapeutic impact in the clinic likely due to their heterogeneity (Rannikko and Hollmén, 2024). Thus, strategies to selectively enrich and/or reprogram tumor-inhibiting versus tumor-promoting myeloid subsets may provide new therapeutic avenues. Moreover, the combination of myeloid-targeted therapies with immune checkpoint inhibitors (ICIs)—for example αPD-1, αPD-L1—can offer a promising approach to overcome the limited response rates of ICIs in some solid tumors (Rebaudi et al., 2024).

CD1d belongs to a group of nonclassical antigen-presenting molecules that present lipids to natural killer T (NKT) cells controlling their activation (Mori et al., 2016). While the role of CD1d in lipid presentation is well established, growing evidence supports additional unconventional roles of CD1d, which impact the functions of CD1d-expressing cells (Evans and Barral, 2024; Olszak et al., 2014; Yue et al., 2005). CD1d is constitutively expressed in human and murine myeloid cells, and we have previously shown a cell-intrinsic function for CD1d in the control of myeloid cell immunity (Brailey et al., 2022). Accordingly, CD1d functions as an immune–metabolic switch in myeloid cells, regulating lipid uptake and subsequently controlling responses to Toll-like receptor stimulation (Brailey et al., 2022). Moreover, CD1d intracellular signaling in macrophages inhibits the NLRP3 inflammasome, which in turn regulates gut–blood barrier integrity and intestinal inflammation (Cui et al., 2020). Added to this, ligation of CD1d (with cross-linking antibodies) on the surface of human peripheral blood monocytes and DCs or murine splenocytes is sufficient to induce secretion of IL-12 in a process mediated by nuclear factor κ-light-chain-enhancer of activated B cell signaling (Teng et al., 2009; Yue et al., 2005). Within the TME, myeloid cells, particularly TAMs, have been shown to express CD1d in solid tumors such as neuroblastoma (Song et al., 2009), ovarian cancer (Li et al., 2023), or murine prostate cancer (Cortesi et al., 2018). While much work has been done regarding the functions of NKT cells within tumors (Godfrey et al., 2018; Terabe and Berzofsky, 2018), whether intrinsic CD1d controls the polarization, function, or heterogeneity of myeloid cells in the TME remains unexplored.

In this manuscript, we have investigated the role of CD1d in controlling the myeloid cell landscape in the TME. We found that CD1d-deficient mice are more resistant to tumor growth than WT mice in models of breast cancer. CD1d deficiency was associated with alterations in macrophage/monocyte subsets, accumulation of DCs and NK cells, and activation of CD8^+^ T cells. The effect of CD1d on myeloid cells is cell-intrinsic, as it can be recapitulated in mixed bone marrow (BM) chimeras, as well as in *in vitro* polarization experiments. These observations prompted us to ask whether CD1d blockade could also control antitumor immunity. We showed that αCD1d antibody controlled the polarization of murine and human macrophages *in vitro*. Moreover, administration of αCD1d to tumor-bearing mice blunted tumor growth, altered the myeloid landscape, and enhanced the efficacy of anti-PD-1 immunotherapy. T cells, NK cells, and IFN-γ contributed to support the anti-CD1d–dependent control of tumor growth. Analyses of myeloid cells in the TME by single-cell RNA sequencing (scRNA-seq) showed that CD1d deficiency triggered changes in the myeloid transcriptional program, leading to an increase in the inflammatory program of *Cxcl10*^+^ inflammatory monocytes, which accumulated in the CD1d-deficient TME in a process dependent on type I IFNs. Critically, we found that inflammatory monocytes are present in human breast cancer, and we define a murine gene signature associated with improved outcomes and response to immunotherapy in cancer patients. Thus, targeting CD1d in the TME could provide a promising avenue to enhance the efficacy of ICIs in cancer treatments.

## Results and discussion

### CD1d controls tumor growth and tumor immune infiltrates

**Figure S1.**
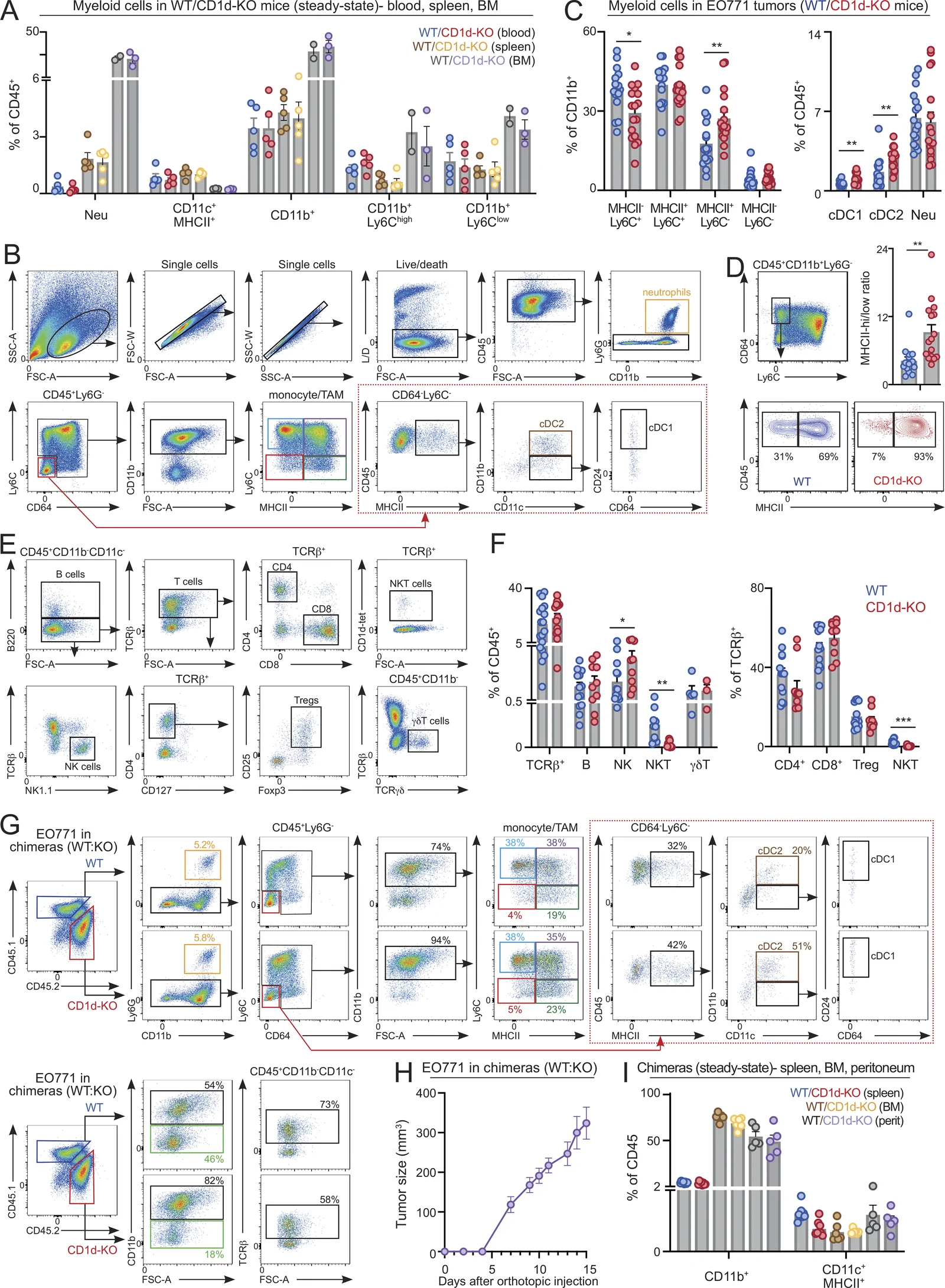
**Immune infiltrates in EO771 tumors in WT and CD1d-KO mice. (A)** Flow cytometry analyses showing frequencies for the depicted myeloid populations in the blood, spleen, and BM from WT and CD1d-KO mice in steady state (*n* = 2–5; data are pooled from two independent experiments). Data are shown as the mean ± SEM. **(B)** Flow cytometry plots showing gating strategy for myeloid cell populations from EO771 tumors. **(C)** Flow cytometry analyses of immune infiltrates in EO771 tumors (from WT and CD1d-KO mice) at day 15 after tumor cell injection, depicting frequencies of the indicated cell populations (*n* = 20; data are pooled from seven independent experiments). Data are shown as the mean ± SEM. **(D)** Flow cytometry plots showing gating strategy and ratio (top, right) of MHCII-hi versus MHCII-low CD64^+^ cells (CD45^+^CD11b^+^CD64^+^Ly6C^−^Ly6G^−^) in WT and CD1d-KO TMEs (*n* = 20; data are pooled from seven independent experiments). Data are shown as the mean ± SEM. **(E)** Flow cytometry plots showing gating strategy for lymphoid cell populations from EO771 tumors. **(F)** Flow cytometry analyses of immune infiltrates in EO771 tumors (from WT and CD1d-KO mice) at day 15 after tumor cell injection, depicting frequencies of the indicated cell populations (*n* = 4–14; data are pooled from three to five independent experiments). Data are shown as the mean ± SEM. **(G and H)** Mixed BM chimeras (WT:CD1d-KO; 50:50) were generated by cotransferring WT (CD45.1^+^) and CD1d-KO (CD45.2^+^) BM into irradiated recipients (CD45.1^+^CD45.2^+^). After reconstitution, mice were orthotopically injected with EO771 cells and immune infiltrates were analyzed at day 15. **(G)** Flow cytometry plots showing gating strategy for the depicted cell populations. **(H)** EO771 tumor growth in chimeras (*n* = 8; data are pooled from two independent experiments). **(I)** Flow cytometry analyses showing frequencies for the depicted cell populations in the spleen, BM, and peritoneal cavity from mixed BM chimeras (WT:CD1d-KO; 50:50) in steady state (no tumor injection, *n* = 5; data are pooled from two independent experiments). Data are shown as the mean ± SEM. *P < 0.05, **P < 0.01, ***P < 0.001, unpaired *t* test.

**Figure 1.**
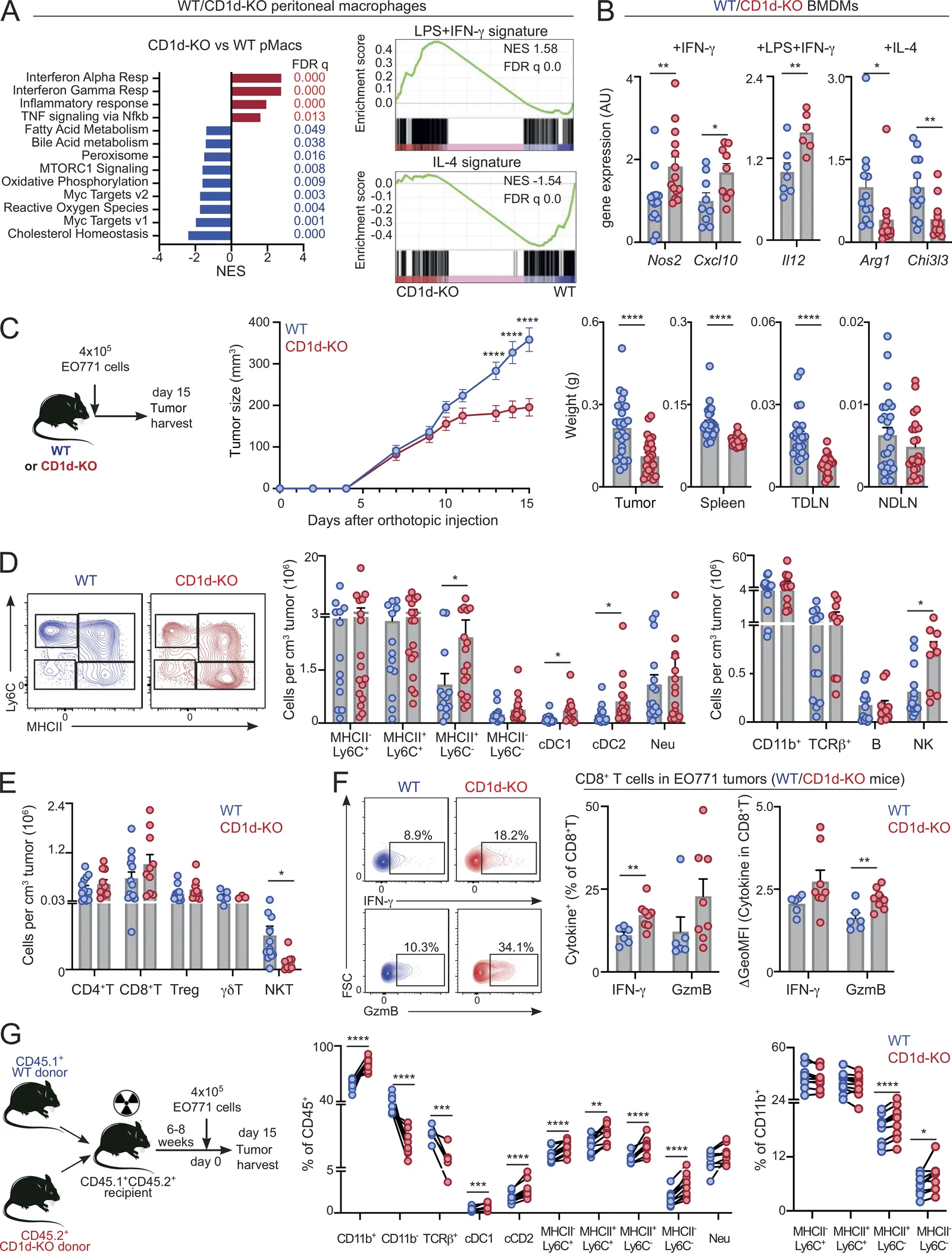
**CD1d deficiency remodels the immune infiltrates in the TME in a manner that supports tumor control. (A)** Bulk RNA-seq for pMacs isolated from WT or CD1d-KO mice. Left: Results of GSEA Hallmark pathway analysis showing top enriched gene sets. NES values indicate enrichment (red bars, positive NES) in CD1d-KO or WT pMacs (blue bars, negative NES). Right: Enrichment plot for transcriptional signature of CD1d-KO (versus WT) pMacs compared with signatures from macrophages stimulated with LPS + IFN-γ (top) or IL-4 (bottom) (Jablonski et al., 2015). **(B)** WT or CD1d-KO BMDMs were stimulated with IFN-γ, IFN-γ and LPS, or IL-4 as indicated, and the expression of the depicted genes was measured by qPCR (*n* = 6–14; data are pooled from 3 to 10 independent experiments). **(C)** WT and CD1d-KO mice were orthotopically injected with EO771 cells, and tumor growth was monitored over time (left). Bar plots represent weights of tumors, spleens, TDLNs, and NDLNs in WT and CD1d-KO mice (*n* = 25; data are pooled from eight independent experiments). Data are shown as the mean ± SEM. **(D and E)** Analyses of immune infiltrates in EO771 tumors from WT or CD1d-KO mice at day 15 after tumor cell injection depicting cell numbers for the indicated cell populations (*n* = 9–20; data are pooled from three to seven independent experiments). Data are shown as the mean ± SEM. **(F)** Representative plots (left) and quantification (right) of frequencies and GeoMFI for IFN-γ^+^ and GzmB^+^ within CD8^+^ T cells in WT and CD1d-KO tumors (day 15) (*n* = 6–8; data are pooled from three independent experiments). Data are shown as the mean ± SEM. **(G)** Mixed BM chimeras (WT:CD1d-KO; 50:50) were generated by cotransferring WT (CD45.1^+^) and CD1d-KO (CD45.2^+^) BM into irradiated recipients (CD45.1^+^CD45.2^+^). After reconstitution, mice were orthotopically injected with EO771 cells and immune infiltrates were analyzed at day 15. Data show frequencies of the WT and CD1d-KO depicted populations in the TME as frequency of CD45 (left) or CD11b (right) (*n* = 8; data are pooled from two independent experiments). *P < 0.05, **P < 0.01, ***P < 0.001, ****P < 0.0001, unpaired (B–F) or paired (G) *t* test or two-way ANOVA with Sidak’s multiple comparisons (C, tumor growth). TDLNs, tumor-draining lymph nodes; NDLNs, nondraining lymph nodes; NES, normalized enrichment score; GeoMFI, geometric mean fluorescence intensity.

Beyond their well-known role in presenting lipid antigens to NKT cells, CD1d molecules also have intrinsic functions, regulating the activation of CD1d-expressing myeloid cells (Evans and Barral, 2024). However, because much of the work exploring the function of CD1d/NKT cells *in vivo* has relied on CD1d-deficient mice—which lack both CD1d and NKT cells—it has been difficult to distinguish the contributions of NKT cells from those of intrinsic CD1d itself. As a result, it remains unknown whether intrinsic CD1d governs the polarization, function, or heterogeneity of myeloid cells in tissues. To start to investigate the role of CD1d/NKT cells in modulating the myeloid cell compartment, we analyzed myeloid cell populations in WT and CD1d-KO animals at the steady state (Fig. S1 A). These analyses did not identify noticeable changes in the frequencies of myeloid cells in BM, blood, or spleen (Fig. S1 A), suggesting that CD1d/NKT cells do not directly regulate myeloid cell frequencies in homeostasis. We further investigated macrophage functions by taking advantage of bulk RNA-seq data obtained from peritoneal macrophages (pMacs) isolated from WT or CD1d-KO mice in the steady state (Brailey et al., 2022). We performed gene set enrichment analyses (GSEAs) for these datasets, which revealed enrichment of pathways related to inflammation and IFN responses in CD1d-KO macrophages, including IFN-α and IFN-γ responses (Fig. 1 A). Moreover, analyses comparing the WT/CD1d-KO transcriptomic datasets with gene signatures for macrophages either classically (LPS + IFN-γ) or alternatively (IL-4) activated (Jablonski et al., 2015) revealed a positive enrichment for classically activated signatures and a negative enrichment for alternatively activated signatures in CD1d-KO cells (Fig. 1 A). This suggests that CD1d-deficient cells present a proinflammatory phenotype, yet we cannot discard that NKT cells may directly or indirectly influence the transcriptional program of pMacs *in vivo*. Thus, to functionally test the role of CD1d in controlling macrophage reprogramming we generated BM-derived macrophages (BMDMs) from WT and CD1d-KO mice, which were stimulated with IFN-γ, IFN-γ + LPS, or IL-4. As predicted by the RNA-seq data, CD1d-KO cells showed an increased expression in IFN-γ–induced genes (*Nos2, Cxcl10, Il-12*) and a decrease in IL-4–induced genes (*Arg1*, *Chi3l3*) (Fig. 1 B). These data indicate a direct role of CD1d in controlling macrophage functional polarization with CD1d-deficient cells showing a proinflammatory phenotype.

To investigate the relevance of the CD1d-dependent regulation of macrophage functional polarization, we established a murine model of breast cancer by orthotopic injection of tumor cells (EO771, derived from a spontaneous mammary tumor in C57BL/6 mouse [Casey et al., 1951]) in the mammary fat pad of WT and CD1d-KO mice (Fig. 1 C). Lack of CD1d/NKT cells led to a strong decrease in tumor size and weight, which became apparent as early as 10 days after cancer cell implantation. Weights of spleens and tumor-draining lymph nodes—but not nondraining lymph nodes—were also significantly reduced in CD1d-KO tumor-bearing mice (Fig. 1 C), indicating that CD1d/NKT cell deficiency supports tumor control. Next, we investigated the immune infiltrates in EO771 tumors from WT and CD1d-KO animals. We analyzed myeloid cells in the TME 15 days after tumor cell injection and defined myeloid cell populations by flow cytometry as depicted in Fig. S1 B. This enabled identification of neutrophils (CD45^+^CD11b^+^Ly6G^+^), conventional DC populations—including cDC1 (CD45^+^CD11c^+^CD11b^−^Ly6C^−^CD64^−^MHCII^+^CD24^+^) and cDC2 (CD45^+^ CD11c^+^CD11b^+^Ly6C^−^CD64^−^MHCII^+^)—and various monocytes/TAM subsets (CD45^+^CD11b^+^Ly6C^+/−^CD64^+/−^), which were distinguished based on the expression of Ly6C and MHCII (Fig. S1, B–D; and Fig. 1 D) (Geeraerts et al., 2021; Movahedi et al., 2010). In the monocyte/TAM populations, we detected an increase in frequency and numbers of MHCII^+^Ly6C^−^ cells (Fig. 1 D and Fig. S1 C). Previous reports proposed that MHCII discriminates immunostimulatory (MHCII-high) versus immunosuppressive (MHCII-low) TAMs (Clark et al., 2025; Lam et al., 2021), and following this approach, we found that within CD64^+^ cells, MHCII^+^ cells became dominant in the CD1d-KO TME (MHCII-hi/MHCII-lo ratio, Fig. S1 D). This suggests an accumulation of proinflammatory cells in CD1d-KO tumors, in keeping with the proinflammatory profile of CD1d-KO myeloid cells detected *in vitro* (Fig. 1, A and B). In addition to monocyte/macrophage changes, conventional DC populations (both cDC1 and cDC2) were also increased in frequency and numbers in CD1d-KO tumors (Fig. 1 D and Fig. S1 C). This myeloid remodeling was accompanied by alterations in lymphoid cell populations in CD1d-KO TMEs (Fig. 1, D and F; and Fig. S1, E and F). CD1d-KO tumor infiltrates contained comparable numbers of T cells (CD4^+^, CD8^+^, regulatory T cells, γδT cells) and B cells to WT infiltrates (Fig. 1, D and E; and Fig. S1 F) but intratumoral NK cell numbers and frequency (Fig. 1 D and Fig. S1 F), and CD8^+^ T cell effector functions (IFN-γ, granzyme B, Fig. 1 F) were increased in the CD1d-KO TME, in line with the more robust tumor control in CD1d-KO mice. Altogether, these data indicate that lack of CD1d/NKT cells remodels the immune infiltrates in the TME in a manner that supports tumor control.

Next, we investigated how the tumor myeloid landscape is regulated by CD1d. CD1d-KO mice lack CD1d expression, as well as NKT cells, which could control the myeloid populations in the TME. Indeed, we detected small numbers of NKT cells in the WT TME, which were absent, as expected, in CD1d-KO animals (Fig. 1 E and Fig. S1 F). Thus, to explore whether the differences in myeloid cells in the TME between CD1d-deficient and CD1d-sufficient mice are cell-intrinsic, we generated mixed BM chimeras (WT:CD1d-KO; 50:50) by cotransferring WT (CD45.1^+^) and CD1d-KO (CD45.2^+^) BM into irradiated recipients (CD45.1^+^CD45.2^+^). After reconstitution, chimeric mice were injected with EO771 cells, and WT/CD1d-KO immune cells were identified in the TME on the basis of their congenic marker expression (Fig. 1 G and Fig. S1, G and H). In this setting, both CD1d-sufficient and CD1d-deficient cells are exposed to the same TME. In these tumors, we noted that CD11b^+^ cells were significantly more abundant within the CD1d-KO population, suggesting that CD1d-KO CD11b^+^ cells may be more efficiently recruited to (and/or retained in) the TME (Fig. 1 G). On the other hand, there was a decrease in the frequency of CD11b^−^ cells in the CD1d-KO compartment, driven primarily by a decrease in the frequency of T cells (Fig. 1 G). Moreover, and consistent with the results observed in the CD1d-KO TME, we measured increased frequencies of MHCII^+^Ly6C^−^ cells (within CD11b^+^ cells), as well as cDC1 and cDC2 within the CD1d-KO compartment (Fig. 1 G). The enrichment in CD1d-KO myeloid cells (CD11b^+^, DCs) was not observed in tissues (spleen, BM, peritoneal cavity) from mixed chimeras in the absence of tumors (Fig. S1 I). Altogether, these data indicate that within the same TME, the CD1d-KO myeloid populations are distinct to those of WT origin suggesting an intrinsic role of CD1d in controlling the tumor immune infiltrates.

Altogether, these data indicate that CD1d contributes to the control of the macrophage proinflammatory status and myeloid subsets in the TME, illustrating a previously unidentified axis for immune regulation mediated by CD1d with direct relevance for antitumor immunity.

### Anti-CD1d treatment reshapes intratumoral myeloid infiltrates, controls tumor progression, and enhances anti-PD-1 immunotherapy efficacy

**Figure 2.**
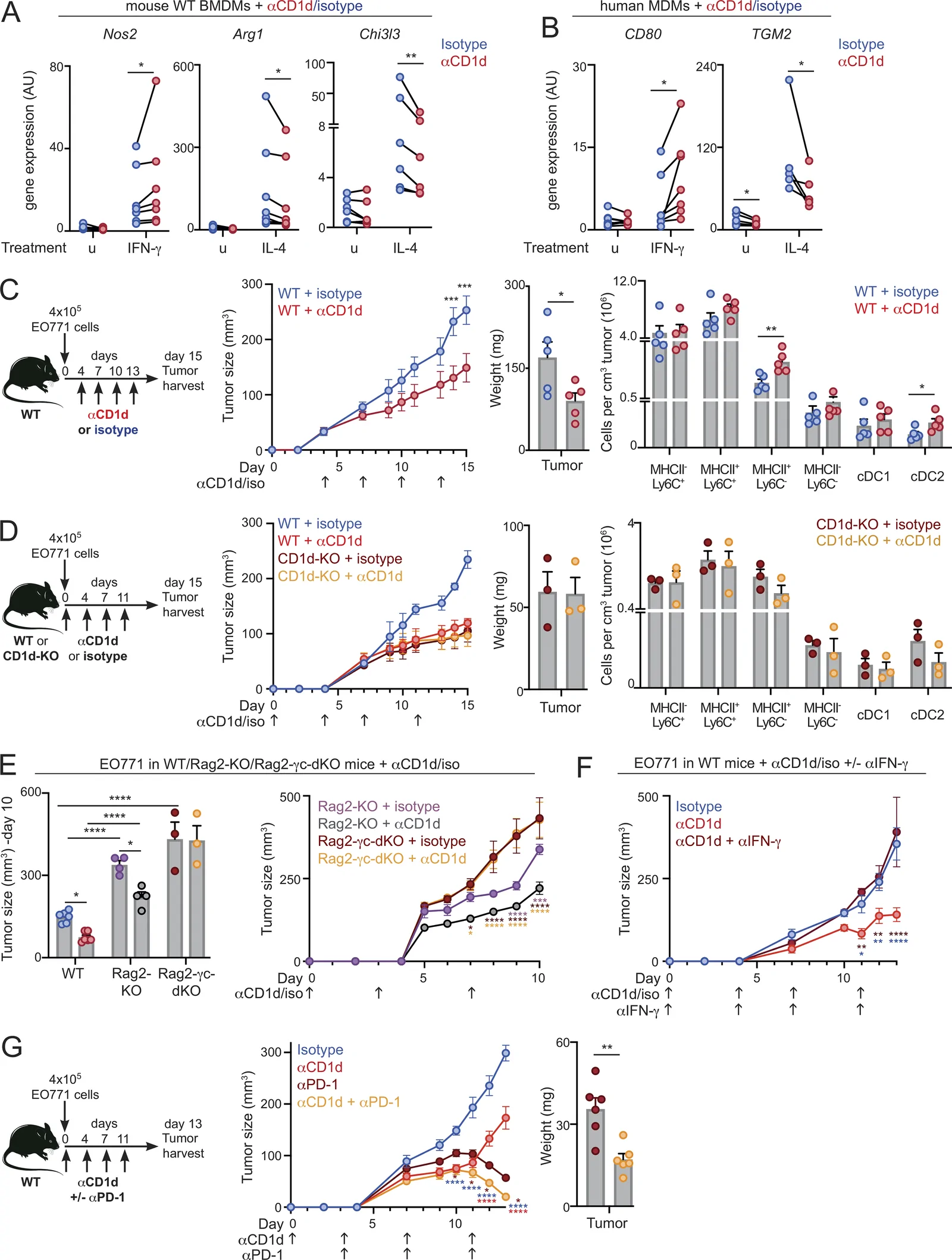
**Anti-CD1d reduces tumor growth and enhances anti-PD-1–induced tumor regression. (A and B)** Mouse BMDMs (A) or human MDMs (B) were cultured with αCD1d or isotype and stimulated with IFN-γ or IL-4 as indicated (or left unstimulated, u). Expression of the depicted genes was measured by qPCR (*n* = 5–7; data are pooled from three independent experiments). **(C)** WT mice were orthotopically injected with EO771 cells and received αCD1d (or isotype) at the indicated time points (arrows). Tumor growth was monitored over time (left). Bar plots represent tumor weight (middle) and numbers for the indicated cell populations (right) at day 15 (*n* = 5; data are pooled from two independent experiments). Data are shown as the mean ± SEM. **(D)** WT or CD1d-KO mice were orthotopically injected with EO771 cells and received αCD1d (or isotype) at the indicated time points (arrows). Tumor growth was monitored over time (left). Bar plots represent tumor weight (middle) and numbers for the indicated cell populations (right) at day 15 (*n* = 3; data are pooled from two independent experiments). Data are shown as the mean ± SEM. **(E)** Tumor size (left, day 10) and growth (right) in WT, Rag2-KO, or Rag2-γc-dKO mice orthotopically injected with EO771 cells and receiving αCD1d (or isotype) at the indicated time points (arrows) (*n* = 3–7; data are pooled from two to three independent experiments). Data are shown as the mean ± SEM. **(F)** Tumor growth in WT mice orthotopically injected with EO771 cells and receiving αCD1d ± αIFN-γ at the indicated time points (arrows) (*n* = 4–7; data are pooled from three independent experiments). Data are shown as the mean ± SEM. **(G)** WT mice were orthotopically injected with EO771 cells and received αCD1d ± αPD-1 (or isotype) at the indicated time points (arrows). The bar plot represents tumor weight at day 12 (*n* = 6; data are pooled from two independent experiments). Data are shown as the mean ± SEM. *P < 0.05, **P < 0.01, ***P < 0.001, ****P < 0.0001, unpaired (C, D, and G) or paired (A and B) *t* test, or two-way ANOVA with Tukey’s (E, left) or Sidak’s (C–G, tumor growth plots) multiple comparisons.

Since genetic ablation of CD1d controls the myeloid landscape and impairs tumor growth, we sought to test the therapeutic potential of monoclonal antibody modulation of CD1d. To investigate whether αCD1d regulates the functional polarization of macrophages, we cultured WT BMDMs with a monoclonal αCD1d blocking antibody (19G11 [Brailey et al., 2022]) or isotype control prior to stimulation with IFN-γ or IL-4. αCD1d treatment recapitulated the phenotype of CD1d-KO cells resulting in the increased expression of IFN-γ–induced genes and a decrease in IL-4–induced genes (Fig. 2 A). Importantly, the αCD1d effect was conserved in human macrophages. Treatment of human monocyte-derived macrophages (MDMs)—generated from healthy donor PBMCs—with αCD1d also increased the expression of IFN-γ–induced genes (*CD80*) and reduced IL-4–induced genes (*TGM2*) (Fig. 2 B). Thus, CD1d controls macrophage polarization in human and murine cells.

**Figure S2.**
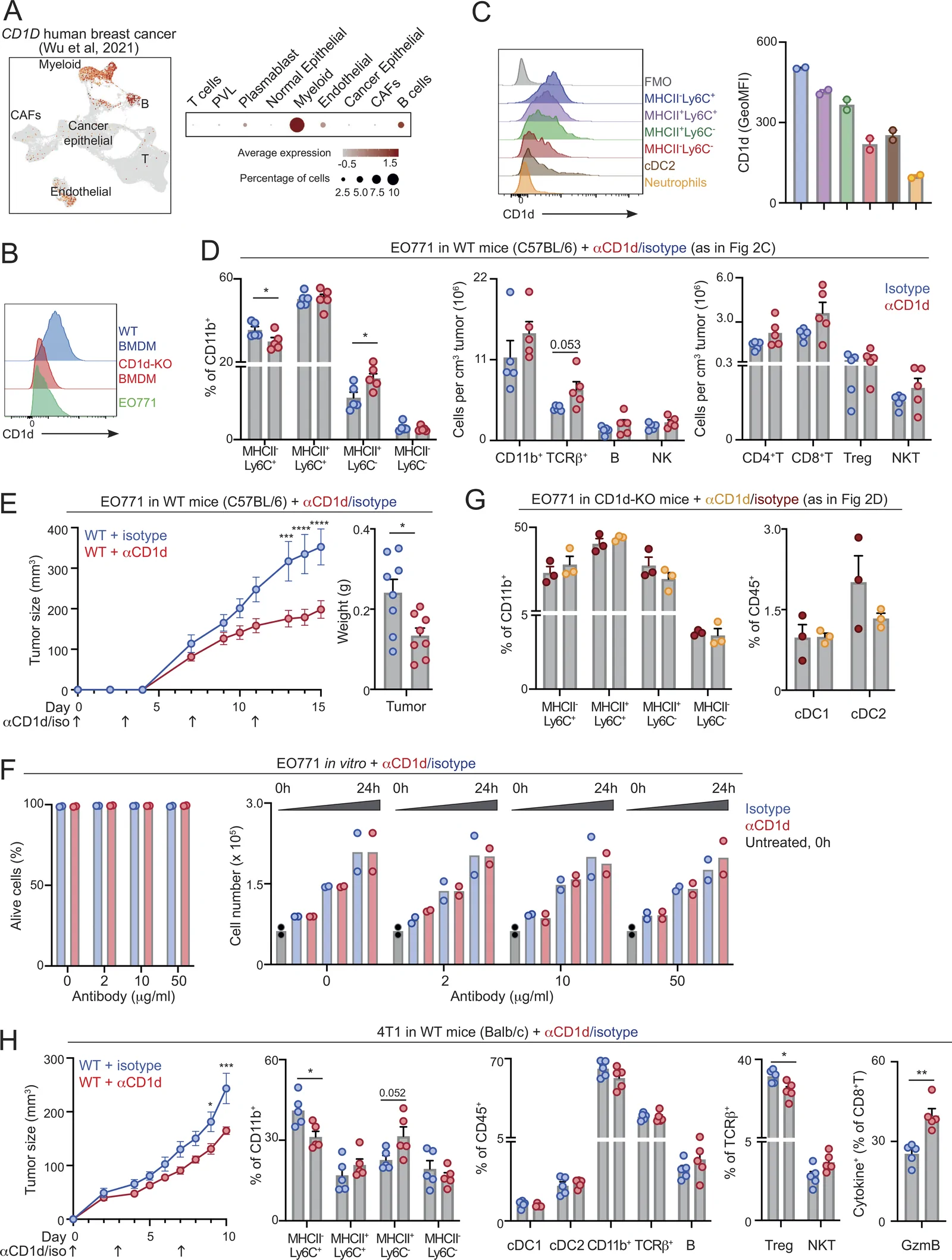
**Anti-CD1d regulates immune infiltrates and tumor growth. (A)** Left: UMAP showing *CD1D* expression in cell clusters from human breast cancers (Wu et al., 2021). Right: Dot plot showing the expression of *CD1D* in the depicted populations of human breast cancers. **(B)** Flow cytometry plot depicting CD1d expression in WT and CD1d-KO BMDMs and in EO771 cells as indicated. Data are representative of two independent experiments. **(C)** Flow cytometry plot for CD1d expression in the depicted myeloid cell populations identified in the EO771 TME in WT mice. Right: GeoMFI for the depicted populations. Data are representative of two independent experiments. **(D)** Frequencies of the indicated cell populations in EO771 tumors in WT mice receiving αCD1d (or isotype) as indicated in Fig. 2 C. **(E)** Tumor growth in WT mice orthotopically injected with EO771 cells and receiving αCD1d (or isotype) at the indicated time points (arrows). Data are shown as the mean ± SEM. The bar plot represents tumor weight at day 15 (*n* = 8; data are pooled from three independent experiments). **(F)** EO771 cells were cultured *in vitro* with increasing concentrations of αCD1d (or isotype) for the indicated time points. Graphs show frequency of alive cells after 24 h (left) and number of cells (right) at different time points after αCD1d culture. Data are representative of two independent experiments. **(G)** Frequencies of the depicted cell populations in EO771 tumors from CD1d-KO mice receiving αCD1d (or isotype) at the time points indicated in Fig. 2 D. Data are shown as the mean ± SEM. **(H)** Tumor growth in WT BALB/c mice orthotopically injected with 4T1 cells and receiving αCD1d (or isotype) at the indicated time points (arrows). Bar plots represent frequencies of the indicated cell populations at day 10 (*n* = 5; data are pooled from two independent experiments). Data are shown as the mean ± SEM. *P < 0.05, **P < 0.01, ***P < 0.001, ****P < 0.0001, unpaired (D, E, and H) *t* test or two-way ANOVA with Sidak’s (E, H, tumor growth plot) multiple comparisons. GeoMFI, geometric mean fluorescence intensity.

Using published scRNA-seq datasets from human breast cancer (Wu et al., 2021), we found that *CD1D* was primarily expressed in myeloid (but not tumor) cells (Fig. S2 A). In keeping with this, murine EO771 breast cancer cells also lacked CD1d expression (Fig. S2 B), while in the TME, myeloid cells express CD1d to various degrees (Fig. S2 C). Thus, we hypothesized that αCD1d injection *in vivo* may target CD1d^+^ cells in the TME, alter the myeloid cell landscape, and contribute to tumor control. We injected tumor-bearing mice with a monoclonal αCD1d antibody (19G11 or isotype control) starting either at day zero (at the same time as tumor cell injection) or at day 4 (as tumors became palpable) followed by injections every 3–4 days as indicated in Fig. 2 C and Fig. S2 E (injection times indicated with arrows). Treatment with αCD1d afforded significant control of tumor growth when administered at both day zero and day 4, and this was accompanied by an increase in MHCII^+^Ly6C^−^ cells and cDC2s (Fig. 2 C and Fig. S2 D), comparable to the myeloid cell rewiring detected in CD1d-KO mice (Fig. 1 D). Importantly, αCD1d treatment did not affect the viability or proliferation of EO771 cells *in vitro* (Fig. S2 F) and it had no effect on tumor growth or immune infiltrates in tumors implanted into CD1d-KO animals (Fig. 2 D and Fig. S2 G), indicating that αCD1d acts on CD1d^+^ cells in the TME and has no observed off-target effects. Moreover, αCD1d also controlled tumor growth and myeloid populations in 4T1 breast tumors injected in the mammary fat pad of WT BALB/c mice (Fig. S2 H), confirming an effective role of αCD1d across different tumor models and murine genetic backgrounds. Of note, αCD1d treatment did not alter the numbers or frequencies of NKT cells in the TME in the EO771 (Fig. S2 D) or 4T1 (Fig. S2 H) models, supporting that its effects are on the CD1d^+^ myeloid cell in the TME.

To explore the mechanisms of αCD1d-mediated tumor control, we injected αCD1d (or isotype) into tumor-bearing Rag2-KO or Rag2-γc double-KO (Rag2-γc-dKO) mice (Fig. 2 E). As expected, EO771 tumor growth was significantly accelerated in Rag2-KO and Rag2-γc-dKO versus WT mice confirming a role of T cells and NK cells (and/or other innate lymphoid cells) in the control of tumor growth in this model (Fig. 2 E). Administration of αCD1d resulted in reduced tumor growth in Rag2-KO animals, although to a lesser extent than in WT mice (Fig. 2 E). This suggests that while T cells are relevant for the CD1d-mediated tumor control, there must be additional protective effects of anti-CD1d independent of T cells. Conversely, αCD1d had no effect in tumors implanted in Rag2-γc-dKO mice confirming NK cell–dependent antitumor immunity (Fig. 2 E). Considering the roles of T cells/NK cells in the anti-CD1d responses and the enrichment of IFN-γ–producing CD8^+^ T cells in the CD1d-KO TME (Fig. 1 F), we speculated that IFN-γ may contribute to the CD1d-dependent control of tumor growth. Indeed, neutralization of IFN-γ inhibited the ability of anti-CD1d to suppress tumor growth (Fig. 2 F). Collectively, these data indicate that anti-CD1d modulates the TME facilitating control of tumor growth by T cells and NK cells through a mechanism involving IFN-γ.

ICIs (αPD-1, αPD-L1) are widely used in the clinic to treat a variety of tumors and recently approved for treatment in triple negative breast cancer (TNBC) (Schmid et al., 2024; Schmid et al., 2022). Given the T cell contribution to the control of tumor growth in our system, we investigated whether αCD1d could function in synergy with αPD-1 to control tumor growth (Fig. 2 G). We injected tumor-bearing mice with αCD1d ± αPD-1 as indicated in Fig. 2 G. Treatment with αPD-1 was sufficient to confer tumor control in all mice tested, yet this effect was accelerated and enhanced when administered in combination with αCD1d, and the tumor size was significantly reduced by the combination treatment (Fig. 2 G). Our data suggest that targeting CD1d in the TME could provide a promising avenue to enhance antitumor immunity and the efficacy of ICIs.

Altogether, these data demonstrate that antibody-mediated modulation of CD1d drives the remodeling of the myeloid compartment in the TME, controls tumor growth, and enhances αPD-1 immunotherapy efficacy. The mechanism(s) by which CD1d deficiency or anti-CD1d treatment controls myeloid cells in the tumors require(s) further investigation, yet our *in vitro* and *in vivo* (chimeras, Rag2-KO) experiments support a model by which intrinsic CD1d tunes macrophage functional polarization, regulating their proinflammatory state. It is also possible that the lack or blockade of CD1d selectively affects the survival of certain myeloid cell subsets, allowing other subsets to expand, subsequently altering the myeloid landscape. Moreover, and not mutually exclusive, NKT cell–myeloid cell crosstalk *in vivo* (involving TCR-CD1d interactions) may also contribute to the control of the recruitment, retention, and/or differentiation of myeloid cells in the tumors. Finally, it is worth mentioning that while the number of tumor-infiltrating NKT cells is not altered by αCD1d administration, the antibody may inhibit their function *in vivo* and this could potentially contribute to tumor control (Teng et al., 2009). However, since αCD1d prevents tumor growth in Rag2-KO animals, its antitumor function must necessarily be (at least in part) NKT cell–independent.

### CD1d reprograms the tumor myeloid compartment

**Figure 3.**
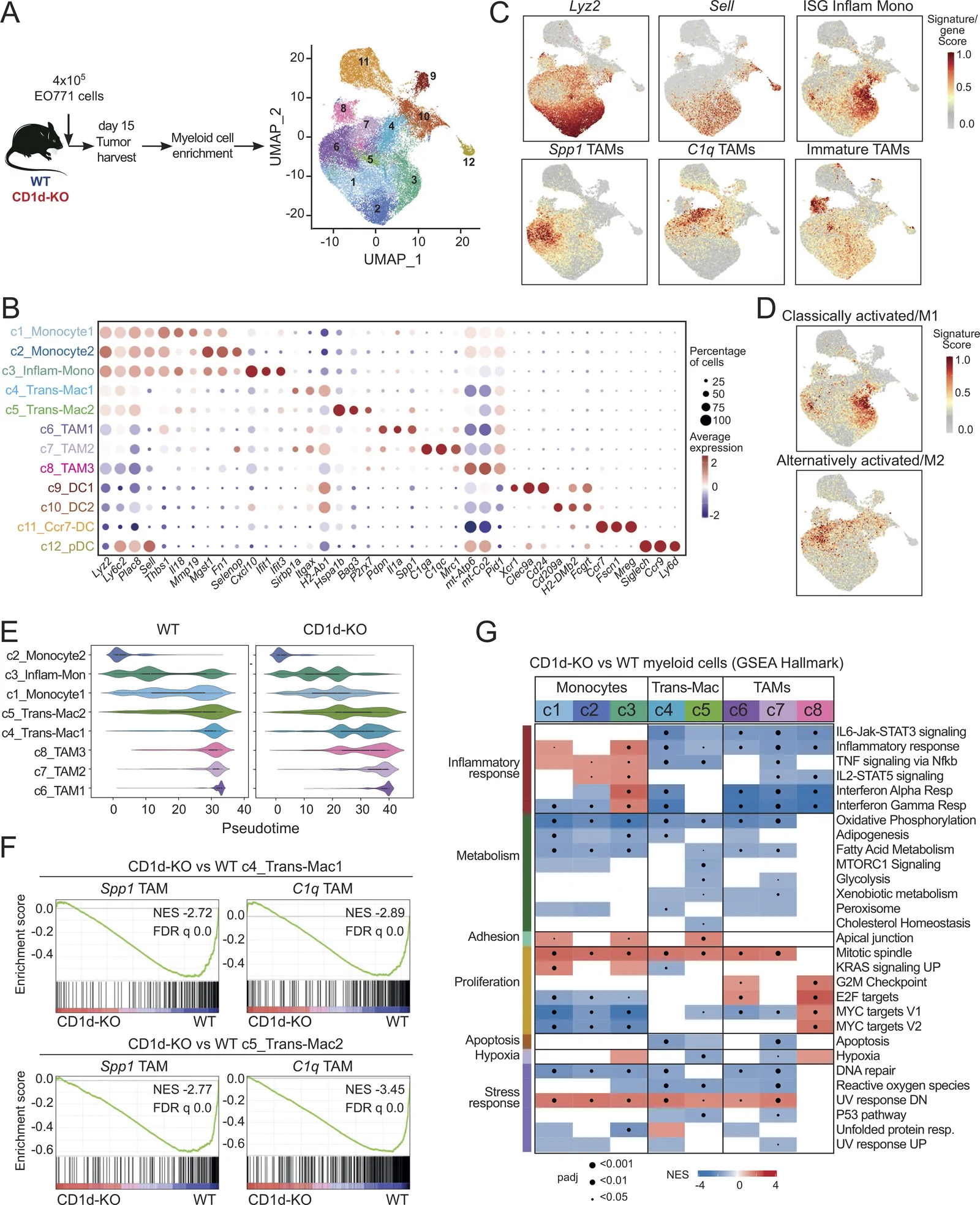
**CD1d controls the myeloid transcriptional program in the TME. (A–G)** scRNA-seq was performed for myeloid cells enriched from EO771 tumors from WT and CD1d-KO mice at day 15 (*n* = 3 mice per condition). **(A)** Experimental setup and UMAP plot of myeloid cell clusters (subsetted for monocytes, TAMs, DCs, and pDCs) from merged WT/CD1d-KO tumors. Each point represents a single cell colored according to cluster designation. **(B)** Dot plot showing expression for selected genes in each of the specified myeloid populations. **(C and D)** Expression levels of selected genes/gene signatures displayed on UMAP plots of myeloid clusters (as in Fig. 3 A). **(E)** Differentiation trajectory model generated by RNA velocity analysis of c1–c8 clusters. Graphs show c1–c8 over pseudotime (ordered by median) estimated from velocity analysis for WT and CD1d-KO samples. **(F)** GSEA enrichment plot for transcriptional signature of CD1d-KO (versus WT) Trans-Mac (c4 [top] and c5 [bottom]) compared with signatures from Spp1 and C1q TAMs. **(G)** GSEA Hallmark pathway analysis showing top enriched signatures. NES values indicate enrichment (red, positive NES) in CD1d-KO or WT cells (blue, negative NES). NES, normalized enrichment score.

**Figure S3.**
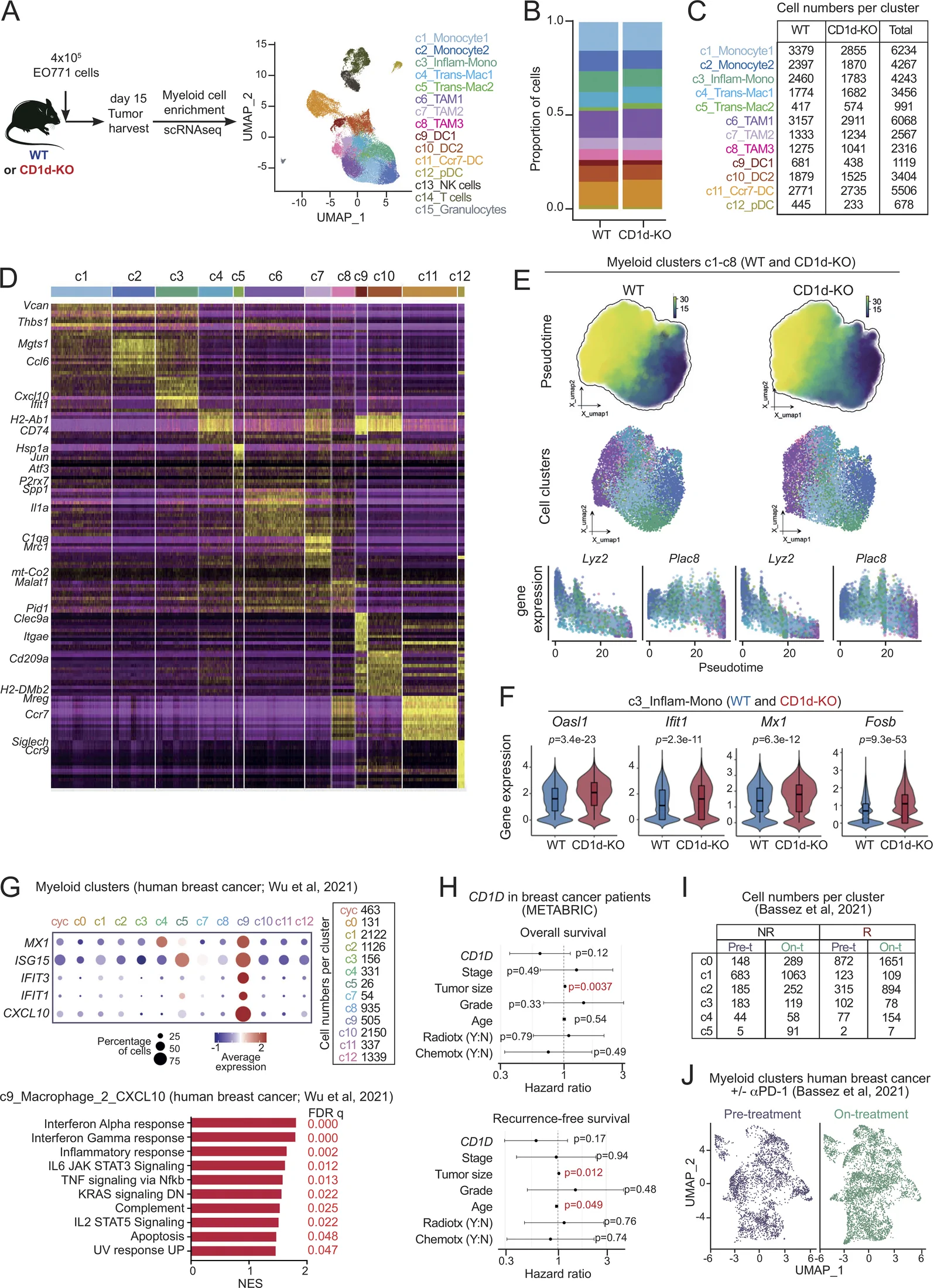
**Transcriptional regulation of myeloid cell populations by CD1d. (A)** Workflow and UMAP plot of cell clusters from merged WT/CD1d-KO tumors. Each point represents a single cell colored according to cluster designation. **(B and C)** Frequencies of cell clusters (B) and number of cells per cluster (C) in WT and CD1d-KO samples. **(D)** Phenotype of the different clusters represented as a heatmap showing the top DEGs per cluster. **(E)** Differentiation trajectory model generated by RNA velocity analysis of c1–c8 clusters for WT and CD1d-KO samples, showing pseudotime (top), cell types (middle), and gene expression (*Lyz2*, *Plac8*) over pseudotime. **(F)** Violin plots showing the expression of the depicted genes in WT and CD1d-KO c3_Inflam-Mono cells. Wilcoxon rank-sum test. **(G)** Top: Dot plot showing expression for selected genes in each of the specified myeloid clusters from human breast cancer (Wu et al., 2021) as depicted in Fig. 5 A (right) and cell numbers per cluster (left). Bottom: GSEA Hallmark pathway analysis showing top positively enriched gene sets in c9_Macrophage_2_CXCL10 macrophages from human breast cancer (Wu et al., 2021). NES = normalized enrichment score. **(H)** Forest plots showing a multivariate Cox regression analysis for the indicated risk factors. **(I and J)** Number of cells per cluster (I) and UMAP plot (J) of myeloid cell clusters from breast cancer patients (Bassez et al., 2021) before (pretreatment) or after (on-treatment) αPD-1 neoadjuvant therapy.

To explore the impact of CD1d on the myeloid cell populations in the TME, we performed scRNA-seq from myeloid cells enriched from EO771 tumors from WT and CD1d-KO mice at day 15 after implantation (*n* = 3 mice per genotype). Unsupervised clustering by UMAP identified 15 clusters from which 12 clusters corresponded to myeloid cells (Fig. 3 A and Fig. S3. A–D). We focused on the myeloid clusters (excluding neutrophils) in which we identified three DC clusters (9–11): c9_DC1, corresponding to cDC1 cells expressing *Xcr1* and *Clec9a*; c10_DC2, corresponding to cDC2 cells expressing *CD209a* (DC-SIGN); and c11_*Ccr7*^*+*^DC, corresponding to activated or so-called mregDCs expressing *Ccr7* and *Mreg* (Ginhoux et al., 2022; Wculek et al., 2020). Moreover, we identified a population of plasmacytoid DCs (c12_pDCs) expressing *Siglech* and *Ly6d* (Fig. 3, A and B). Next, we interrogated the monocyte and TAM clusters. We assessed the expression of canonical markers of monocytes (*Lyz2*, *Ly6c2*, *Plac8*, *Sell*) and mature macrophages (*C1q*, *Mrc1*, *Spp1*) in the monocyte/TAM subpopulations and found that *Lyz2*, *Ly6c2*, *Sell*, and *Plac8* were highly expressed in clusters 1–3, while mature macrophage markers were higher in TAMs (clusters 6–8, Fig. 3, A and B). To functionally characterize the monocyte/macrophage populations, we analyzed these clusters for their scores in signatures associated with populations previously described in the literature, as well as with proinflammatory (classically activated/M1) or immunosuppressive (alternatively activated/M2) signatures (Fig. 3, C and D). Within our dataset, cluster 3 (c3) was the only population enriched for the expression of proinflammatory/M1 signatures and did not display alternatively activated signatures (Fig. 3 D). This cluster was also characterized by the high expression of *Cxcl10* and IFN-inducible genes (ISGs, Fig. 3 B), as well as previously defined ISG signature scores (Duong et al., 2022; Elewaut et al., 2025), defining them as inflammatory monocytes (c3_Inflam-Mono, Fig. 3 C). Clusters 4 (c4) and 5 (c5) showed intermediate expression of *Lyz2*, *Ly6c2*, and *Plac8* but lacked expression of *Sell* and showed low expression of several macrophage markers (*Spp1*, *Mrc1*), which could indicate a transitional state between monocytes and macrophages (Mujal et al., 2022) (Fig. 3, B and C). We called these populations transitioning macrophages (Trans-Mac). On the other hand, TAM clusters 6 (c6_TAM1) and 7 (c7_TAM2) showed the highest enrichment in immunosuppressive signatures and signatures associated with alternatively activated macrophages, and corresponded to previously described immunosuppressive *Spp1* (c6_TAM1) or *C1q* (c7_TAM2) macrophage populations (Jablonski et al., 2015; Zhang et al., 2020) (Fig. 3, C and D). Cluster 8 (c8_TAM3) showed enrichment in signatures of immature macrophages (van Elsas et al., 2024), and high expression of metabolic genes (Fig. 3, C and D).

We next investigated the effect of CD1d in controlling the myeloid populations in the tumors. Inflammatory cues recruit hematopoietic stem cell–derived monocytes that differentiate into TAMs in the TME, and—as tumors grow—these MDMs dominate the macrophage compartment (Casanova-Acebes et al., 2021; Franklin et al., 2014). To further investigate the myeloid populations in EO771 tumors, we generated a model of monocyte-to-macrophage differentiation by computing RNA velocity to the scRNA-seq data for WT and CD1d-KO samples (Fig. 3 E and Fig. S3 E). This method estimates the kinetics of mRNA splicing in single-cell transcriptomes to reconstruct cellular dynamics and infers pseudotime. This model placed c2_Monocyte2 and TAM clusters at opposite ends of the trajectory, while other monocyte and Trans-Mac populations were found in a continuum in agreement with the monocyte-to-macrophage differentiation (Fig. 3 E and Fig. S3 E). Kinetic analysis of cluster-enriched genes confirmed gradual downregulation of monocyte-associated genes (*Plac8*, *Sell*) along the pseudotime trajectory (Fig. S3 E). Interestingly, whereas cells from the WT samples acquired progressively increased pseudotime scores, this progression was delayed for CD1d-KO clusters, as evidenced in monocytes (c1–3) and Trans-Mac (c4 and c5) populations, suggesting that these cells could be more immature (Fig. 3 E). In support of this hypothesis, Spp1 and C1q TAM signatures (Zhang et al., 2020) were enriched in WT versus CD1d-KO Trans-Mac clusters (Fig. 3 F). To further explore the CD1d-dependent regulation of myeloid cells, we performed differential gene expression and GSEA Hallmark pathway analyses to identify enriched pathways controlled by CD1d (Fig. 3 G). Among those, inflammatory pathways (inflammatory response, TNFA signaling, IL-2-Stat 5 signaling) were enriched in CD1d-KO monocytes (c1–3), suggesting that CD1d-KO monocytes preserve a proinflammatory signature in the TME, while these pathways are negatively enriched in immunosuppressive TAMs (c6–8). On the other hand, we found a negative enrichment in CD1d-KO cells of pathways associated with lipid metabolism (fatty acid metabolism, oxidative phosphorylation, peroxisome, mTORC1 signaling), consistent with the immune–metabolic function of CD1d (Brailey et al., 2022).

These data indicate that CD1d-KO monocytes are delayed in their transition to TAMs and preserve a proinflammatory signature in the TME. In addition to the alterations in inflammatory genes, CD1d-KO cells show a negative enrichment in pathways associated with lipid metabolism, recapitulating the transcriptional program observed in CD1d-KO pMacs at the steady state (Fig. 1 A). This suggests that as we previously demonstrated *in vitro* (Brailey et al., 2022), CD1d may function as an immune–metabolic switch controlling the myeloid cell populations in the TME. Although the precise mechanisms by which CD1d regulates this immune–metabolic axis *in vivo* remain to be defined, our *in vitro* data demonstrated that CD1d controls lipid uptake through the scavenger receptor CD36, leading to changes in lipid metabolism and subsequently regulating immune responses (Brailey et al., 2022). We found that this CD1d-CD36 axis is independent on the CD1d intracellular tail, and it can be directly modulated by an anti-CD1d blocking antibody that disrupts CD1d-CD36 interactions recapitulating the metabolic and immune phenotypes of CD1d knockdown (Brailey et al., 2022). On the other hand, and not mutually exclusive, CD1d function could also involve signaling mediated by the CD1d intracellular tail. For example, LPS stimulation has been proposed to initiate intracellular signaling through phosphorylation/dephosphorylation of Tyr/Ser residues in the CD1d intracellular tail ultimately influencing macrophage activation (Cui et al., 2020; Liu et al., 2019). Overall, it is likely that the different stimuli/cytokine milieu associated with specific tissue environments and/or disease states may drive different CD1d-dependent mechanisms, ultimately dictating myeloid cell heterogeneity and functions in the tissues.

### CD1d controls the accumulation and transcriptional program of inflammatory monocytes in breast tumors

**Figure 4.**
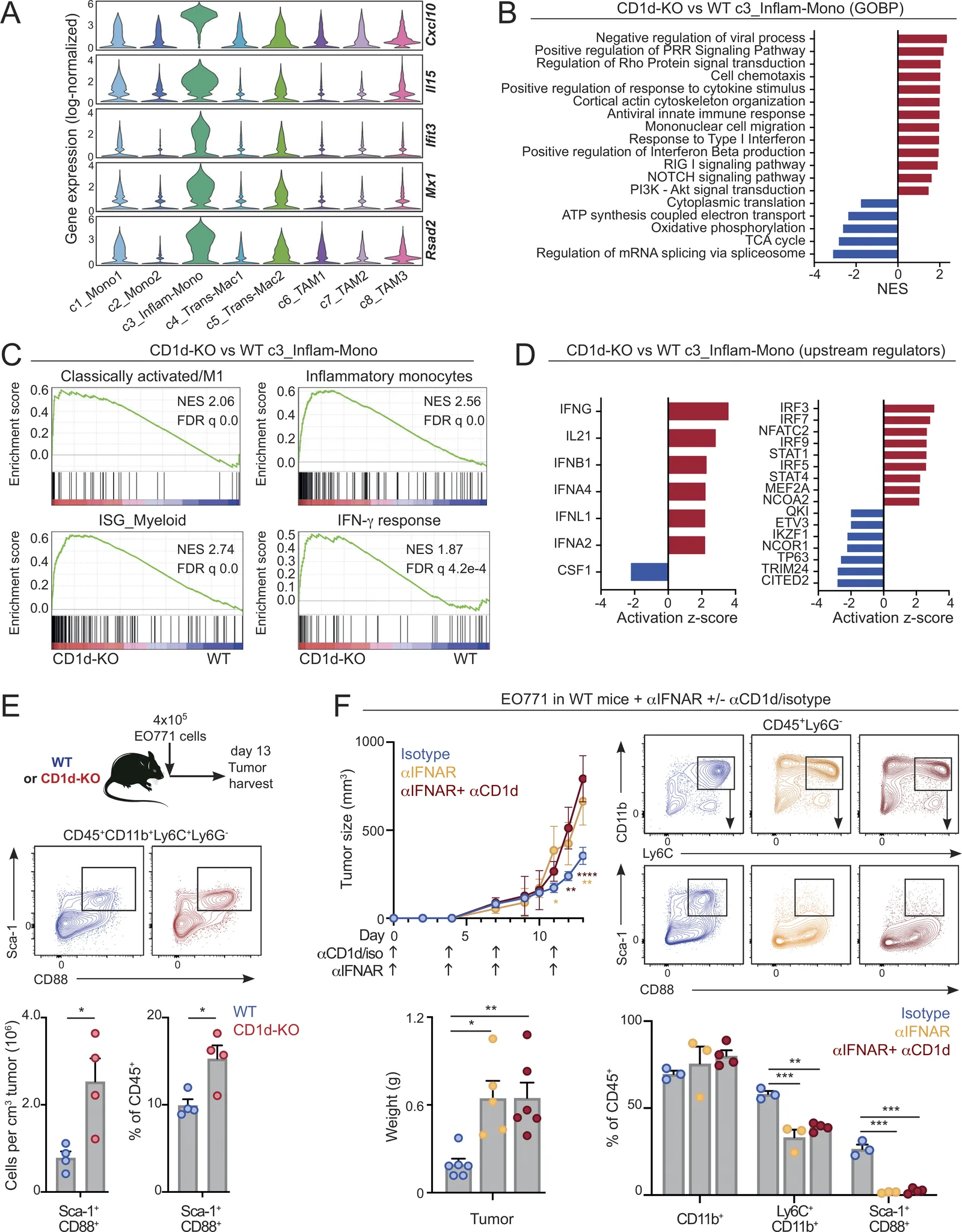
**CD1d controls the transcriptional program and accumulation of inflammatory monocytes. (A)** Violin plots showing the expression of the depicted genes in the myeloid clusters identified by scRNA-seq (as in Fig. 3, A and B). **(B)** GSEAs for GOBP showing top enriched pathways. NES values indicate enrichment (red, positive NES) in CD1d-KO or WT c3_Inflam-Mono cells (blue, negative NES). **(C)** Enrichment plot for transcriptional signature of CD1d-KO versus WT c3_Inflam-Mono cells compared with the depicted signatures. **(D)** Predicted upstream regulators of gene expression changes in CD1d-KO versus WT c3_Inflam-Mono cells, by Ingenuity Pathway Analyses. Data show the top cytokine and transcription factor regulators. z-score indicates the predicted activation level, with either positive (red) or negative (blue) values indicating an activated or inhibited regulator, respectively. **(E)** Experimental setup (top), representative flow cytometry profile (middle), and quantification (bottom) of inflammatory monocytes in EO771 tumors from WT and CD1d-KO mice (*n* = 4; data are pooled from two independent experiments). Data are shown as the mean ± SEM. **(F)** WT mice were orthotopically injected with EO771 cells and received αIFNAR ± αCD1d or isotype at the indicated time points (arrows). Data show tumor growth (top left) and weight (bottom left), as well as flow cytometry plots (top right) and quantification (bottom right) of the depicted myeloid cell populations (*n* = 3–6; data are pooled from three independent experiments). Data are shown as the mean ± SEM. *P < 0.05, **P < 0.01, ***P < 0.001, ****P < 0.0001, unpaired *t* test (E) or one-way or two-way ANOVA with Tukey’s (F) multiple comparisons. GOBP, Gene Ontology Biological Processes; NES, normalized enrichment score.

Given that inflammatory monocytes (c3_Inflam-Mono) were the only population enriched for inflammatory/M1 signatures in our datasets (Fig. 3 D), we investigated whether their immunostimulatory capacity is regulated by CD1d. Inflammatory monocytes have been identified in murine models of melanoma (YUMM1.7) and colon adenocarcinoma clone 38 (MC38), and have been proposed to have immunostimulatory capacity, driving intratumoral T cell stimulation (Elewaut et al., 2025; Kwart et al., 2022). In agreement with published data, c3_Inflam-Mono in our dataset express high levels of ISGs, as well as *Il15*, which mediate T cell survival and effector differentiation (Di Pilato et al., 2021), and *Cxcl9* and *Cxcl10*, which are essential for T cell recruitment and linked to positive immunotherapy responses (Spranger et al., 2017) (Fig. 4 A). Analyses of Gene Ontology Biological Processes pathways for c3_Inflam-Mono (Fig. 4 B) revealed enrichment in pathways related to inflammation and IFN response (including antiviral immune response, response to type I IFN, or positive regulation of response to cytokines) in CD1d-KO versus WT cells, as well as a negative enrichment of metabolic pathways (e.g., oxidative phosphorylation), in keeping with the GSEA Hallmark pathway results (Fig. 3 G). Additionally, signatures associated with ISG+ myeloid cells, M1 macrophage polarization, and IFN-γ response (Bosteels et al., 2020; Duong et al., 2022; Elewaut et al., 2025; Jablonski et al., 2015) were enriched in CD1d-KO versus WT cells (Fig. 4 C). Accordingly, CD1d-KO cells show an increased expression of ISGs (e.g., *Ifit1*, *Ifit2*, *Ifi208*, *Oasl1*, *Mx1*), as well as of genes associated with proinflammatory macrophage responses such as the Rho GTPase *RhoB* or the transcription factors *Fosb* and *Junb* (Fig. S3 F). Analyses of upstream regulators of this transcriptional program identified cytokines such as IFN-γ and IFN-β and transcription factors such as IRF3, IRF7, or STAT1 as key drivers of the CD1d-KO proinflammatory program (Fig. 4 D). On the other hand, the cytokine CSF1—which drives macrophage differentiation *ex vivo* and immunosuppressive macrophage polarization (Chen et al., 2025)—and the transcription factor CITED2—which negatively regulates inflammatory responses in macrophages (Pong Ng et al., 2020)—showed a negative activation z-score, suggesting that these factors/pathways are inhibited in CD1d-KO cells versus WT counterparts (Fig. 4 D).

Given that CD1d controls the transcriptional program of inflammatory monocytes, we investigated the abundance of these cells in the TME. In tumors, murine inflammatory monocytes have been characterized by surface expression of CD88 (C5aR1), as well as the IFN-induced surface marker Ly6A (Sca-1) (Elewaut et al., 2025; Kwart et al., 2022). Our flow cytometry analyses identified an abundant population of inflammatory monocytes within EO771 tumors, expressing Ly6C, CD88, and Sca-1 (Fig. 4 E). Strikingly, we found that inflammatory monocytes are enriched in the CD1d-KO TME (Fig. 4 E), indicating that CD1d controls the accumulation and transcriptional program of these cells. Next, we investigated the signals driving inflammatory monocyte accumulation in the tumors. Type I IFNs have been proposed to drive the inflammatory state and differentiation of inflammatory monocytes (Elewaut et al., 2025; Kwart et al., 2022), and IFNs appear as key upstream regulators of the inflammatory program of CD1d-KO cells (Fig. 4 D). Hence, we investigated the functions of type I IFNs in breast tumors by injecting tumor-bearing mice with anti-IFN-α/β receptor (αIFNAR) ± αCD1d or isotype as indicated in Fig. 4 F. EO771 tumor growth was significantly accelerated in response to αIFNAR administration versus isotype-treated mice. Moreover, αIFNAR induced strong changes in the myeloid cell populations, including a decrease in the frequency of monocytes (CD11b^+^Ly6C^+^), and an almost complete ablation of Sca-1^+^CD88^+^ cells. Both tumor growth and myeloid cell populations remained unaffected by coadministration of αCD1d together with αIFNAR (Fig. 4 F), indicating that type I IFNs contribute to the regulation of the CD1d-dependent control of tumor progression.

Altogether, our data demonstrate that CD1d modulates the transcriptional program and accumulation of inflammatory monocytes in breast tumors. The CD1d-dependent transcriptional program and monocyte accumulation appear to be regulated by type I IFNs and related transcription factors such as IRFs. It is possible that the increased responsiveness of CD1d-KO cells to inflammatory stimuli (as evidenced *in vitro*, Fig. 1, A and B) and/or an increased concentration of IFNs in the CD1d-KO TME will favor the accumulation of inflammatory cells supporting tumor control in CD1d-KO animals. While the primary source of type I IFN in EO771 tumors remains unknown, our previous data demonstrated that CD1d-deficient macrophages secrete more IFN-β than their WT counterparts in response to stimulation (Brailey et al., 2022), potentially contributing to an inflammatory environment in the tumors. Moreover, tumor cell–derived type I IFNs have been proposed as key mediators of inflammatory monocyte differentiation in models of melanoma and colon cancer (Elewaut et al., 2025; Kwart et al., 2022). The precise function of inflammatory monocytes in the tumors remains poorly understood, but these cells have been shown to exhibit immunostimulatory capacities and are characterized by the expression of CXCR3 ligands (regulating T cell recruitment), as well as IL-15, which promotes the survival, proliferation, and cytotoxicity of NK and CD8^+^ T cells (Elewaut et al., 2025; Ma et al., 2022). Hence, it is feasible to speculate that the accumulation of inflammatory monocytes in CD1d-KO tumors may contribute to the remodeling of the immune infiltrates in the TME in a manner that supports tumor control.

### A CD1d-KO–associated gene signature correlates with clinical outcomes and response to immunotherapy in breast cancer patients

**Figure 5.**
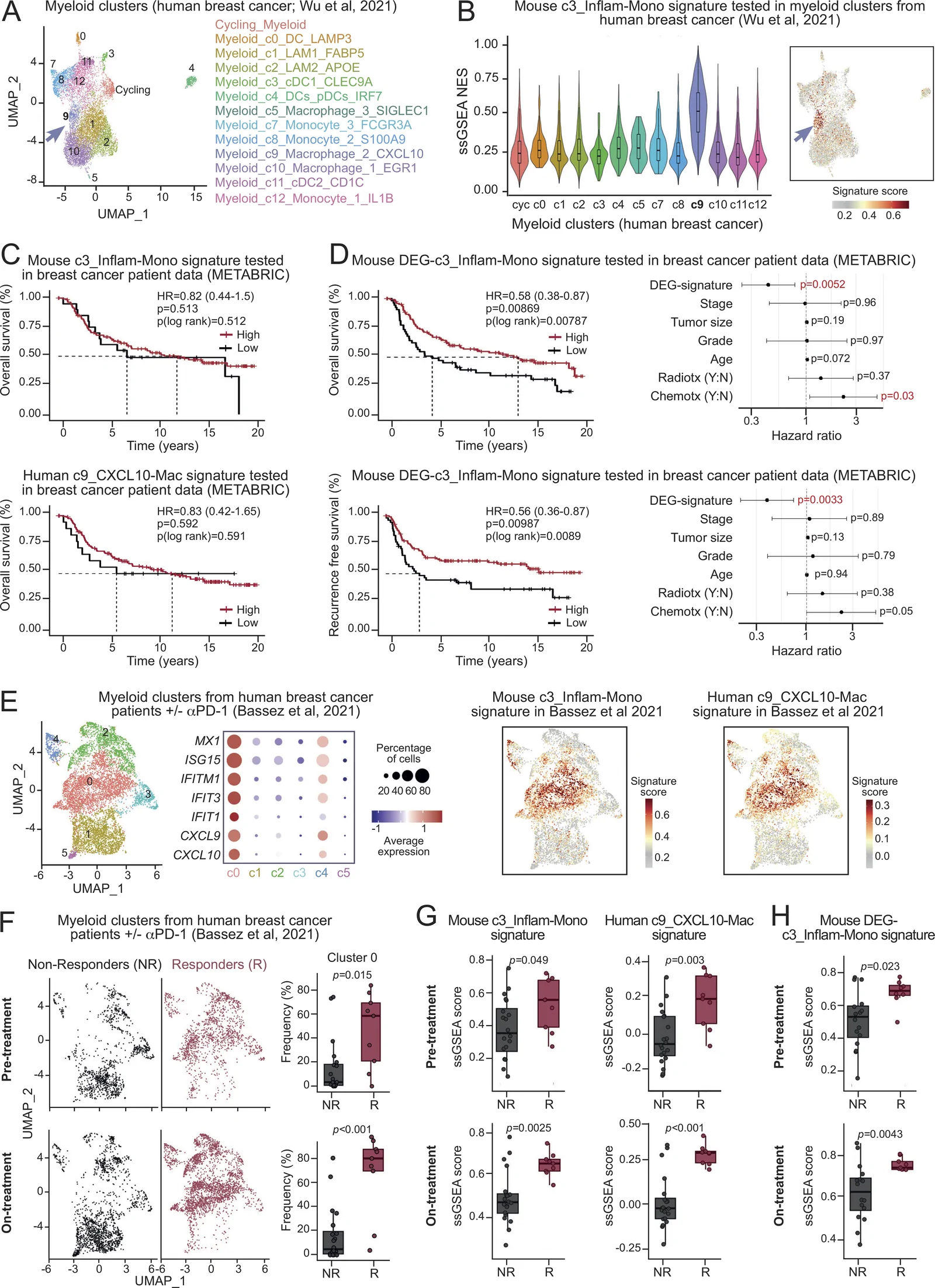
**CD1d-associated gene signature correlates with clinical outcomes and response to immunotherapy in breast cancer patients. (A)** UMAP plot of myeloid cell clusters (filtered for monocytes, TAMs, DCs, and pDCs) from human breast cancers (Wu et al., 2021). Each point represents a single cell colored according to cluster designation. **(B)** Left: Enrichment score for the mouse c3_Inflam-Mono signature in each of the human myeloid clusters (Wu et al., 2021). Right: Expression levels for the murine c3_Inflam-Mono signature displayed on UMAP plot of human myeloid clusters (as in Fig. 5 A). **(C and D)** Survival analyses of TNBC patients (METABRIC), stratified according to mouse c3_Inflam-Mono signature, human c9_CXCL10-Mac signature (C), or mouse DEG- c3_Inflam-Mono signature (D). Kaplan–Meier survival plots parsed as high versus low based on the best cutoff. Log-rank (Mantel–Cox) test. HRs and 95% confidence intervals were estimated using Cox proportional hazards regression. **(D)** Right: Forest plots showing a multivariate Cox regression analysis for the indicated risk factors. HRs with 95% confidence intervals are shown. **(E)** UMAP plot of myeloid cell clusters from breast cancer patients treated with αPD-1 neoadjuvant therapy (Bassez et al., 2021). Each point represents a single cell colored according to cluster designation. Middle: Dot plot showing expression for selected genes in each of the specified myeloid clusters. Right: Expression levels for the mouse c3_Inflam-Mono signature and human c9_CXCL10-Mac signature displayed in the UMAP plot for myeloid cells for human breast cancer patients (Bassez et al., 2021). **(F)** UMAP plot of myeloid cell clusters from breast cancer patients before (pretreatment) or after (on-treatment) αPD-1 neoadjuvant therapy (Bassez et al., 2021). Data are split on patients that respond (R, red) or not (NR, gray) to αPD-1. Bar plots (right) show the frequency of cluster 0 cells in R and NR samples (Wilcoxon rank-sum tests). Each dot is a sample from a different donor. **(G and H)** Enrichment score (ssGSEA) for mouse c3_Inflam-Mono signature and human c9_CXCL10-Mac signature in human myeloid clusters from Bassez et al. (2021) as identified in Fig. 5 E (G) or mouse DEG-c3_Inflam-Mono signature in cluster 0 (H). Data are shown for samples before (pretreatment) or after (on-treatment) αPD-1 neoadjuvant therapy and split for patients that respond (R, red) or not (NR, gray) to αPD-1 (Wilcoxon rank-sum tests). Each dot is a sample from a different donor. HRs, hazard ratios; R, responders; NR, nonresponders.

Next, we investigated whether a comparable population to murine inflammatory monocytes is present in human breast cancer, by interrogating myeloid cell populations from a human scRNA-seq dataset from breast cancer patients (Wu et al., 2021) (Fig. 5 A). We created a murine inflammatory monocyte (c3_Inflam-Mono) gene signature and applied it to human myeloid cell populations from this dataset (Fig. 5 B) (Wu et al., 2021). These analyses revealed a strong overlap between the murine c3_Inflam-Mono signature and that of human cluster 9 corresponding to CXCL10+ macrophages (c9_Macrophage_2_CXCL10) (Fig. 5, A and B). Our GSEAs revealed this human cluster to be enriched for signatures associated with inflammation and IFN response and this is further characterized by high expression of *CXCL10* and ISGs (Fig. S3 G), comparable to their murine counterparts. Thus, ISG+ macrophages with a proinflammatory profile are present in human breast cancer. Interestingly, a similar population of ISG^+^ (CXCL9^+^CXCL10^+^) macrophages has been identified in human melanoma (Elewaut et al., 2025), with cells colocalizing with CD8^+^ T cells and DCs creating inflammatory hubs, which have been linked with positive immunotherapy responses (Chen et al., 2024; Magen et al., 2023).

Next, we investigated whether inflammatory monocytes/CXCL10^+^ macrophages are associated with clinical outcomes of breast cancer patients. To assess the prognostic value of the murine c3_Inflam-Mono signature, we performed univariate and multivariate survival analyses by using Cox proportional hazards regression models taking advantage of data from patients with TNBC obtained from the Molecular Taxonomy of Breast Cancer International Consortium (METABRIC) cohort (Curtis et al., 2012). The stratification of patients according to murine c3_Inflam-Mono signature did not have clinical predictive value in this cohort (Fig. 5 C). Similar results were obtained when using the gene signature derived from the human c9_Macrophage_2_CXCL10 cluster (c9_CXCL10-Mac, Fig. 5 C). A recent report suggested that macrophage polarity in the TME is a better predictor of patient outcomes than TAM numbers (Bill et al., 2023). Thus, we tested whether the CD1d-associated transcriptional program of inflammatory monocytes could correlate with patient outcomes. We derived a gene signature based on the differentially expressed genes (DEGs) upregulated in CD1d-KO versus WT c3_Inflam-Mono in our murine scRNA-seq dataset (DEG-c3_Inflam-Mono signature). The stratification of patients according to DEG-c3_Inflam-Mono signature showed that this signature was associated with beneficial patient outcomes in terms of both overall survival and recurrence-free survival (Fig. 5 D). DEG-c3_Inflam-Mono signature–based patient stratification was independently prognostic when adjusted for age, tumor stage, grade, or chemotherapy/radiotherapy treatment (Fig. 5 D). Of note, *CD1D* expression itself was not associated with patient outcomes (Fig. S3 H). These data suggest that inflammatory monocyte/CXCL10^+^ macrophage polarity correlates with disease outcomes in patients with TNBC.

Finally, we explored whether inflammatory monocyte/CXCL10^+^ macrophages are associated with ICI responses in breast cancer patients. We took advantage of a dataset comprising pre- and posttreatment biopsies for 40 breast cancer patients treated with neoadjuvant αPD-1 (clinical trial NCT03197389) (Bassez et al., 2021). In this dataset, T cell clonotype expansion was used as a surrogate for treatment response. We reanalyzed the myeloid cell populations in these datasets (Fig. 5 E; and Fig. S3, I and J) and identified that our murine c3_Inflam-Mono signature and human c9_CXCL10-Mac signature overlapped with cluster 0 and this cluster expressed high levels of *CXCL9*, *CXCL10*, and ISGs (Fig. 5 E). We observed that neoadjuvant αPD-1 treatment did not induce changes in the total proportions of myeloid cells (Fig. S3, I and J), yet cluster 0 in this dataset (corresponding to CXCL9^+^CXCL10^+^ macrophages) was overrepresented in samples from patients that responded to neoadjuvant αPD-1 versus nonresponders both before and after αPD-1 treatment (Fig. 5 F). In keeping with this, murine c3_Inflam-Mono and human c9_CXCL10-Mac signatures were enriched in pre- and on-treatment tumor samples of patients that had responded to αPD-1 (Fig. 5 G). Moreover, the DEG-c3_Inflam-Mono signature was also enriched in cluster 0 in αPD-1 responders (Fig. 5 H), suggesting that both the abundance and proinflammatory profile of CXCL10+ macrophages correlate with ICI response in breast cancer patients.

Altogether, our data reveal CD1d as a regulator of myeloid cells in the TME. Targeting of CD1d in murine models of breast cancer reduces tumor growth, alters immune cell infiltration, and reprograms tumor-associated myeloid populations toward a proinflammatory state, marked by the expansion of inflammatory monocytes. By integrating murine and human datasets, we identified a population of ISG^+^ (CXCL10^+^) macrophages (equivalent to murine inflammatory monocytes) in human breast tumors, and these cells preferentially accumulate in patients who respond to adjuvant αPD-1 therapy. In addition, we developed a gene signature that correlates with clinical outcomes in breast cancer cohorts. Importantly, blockade of CD1d is sufficient to rewire the myeloid cell populations, reduce tumor growth, and enhance the effectiveness of anti-PD-1 immunotherapy, suggesting an actionable pathway for therapeutic intervention.

## Materials and methods

### Mice and cells

CD1d-KO (B6.129S6-Del(3Cd1d2-Cd1d1)1Sbp/J), WT (C57BL/6, congenic CD45.1 or CD45.1/CD45.2 C57BL/6, and BALB/c), Rag2-KO, and Rag2-γc-dKO mice were bred and maintained in individually ventilated cages under specific pathogen-free conditions at the Francis Crick Institute or King’s College London. All mice were housed under a 12-h light/12-h dark cycle with ad libitum access to food and water, at a temperature of 19–21 °C and humidity of 45–65%. Experiments were carried out using 8- to 16-wk-old mice with age- and gender-matched between genotypes. All animal experiments were approved by the Francis Crick Institute and the King’s College London’s Animal Welfare and Ethical Review Body and the United Kingdom Home Office and performed in accordance with the Animals (Scientific Procedures) Act 1986.

EO771 cells were kindly provided by Dinis Calado (Francis Crick Institute, London, UK). 4T1 cells were obtained from the Francis Crick Institute Cell Services. Murine breast cancer cell lines were maintained in complete DMEM (Gibco; 10% FCS, 100 U/ml penicillin, 100 μg/ml streptomycin, 14.3 μM β-mercaptoethanol) at 37°C in 5% CO_2_.

Healthy human PBMCs were isolated from leukocyte cones purchased from the NHS Blood and Transplant (UK) and obtained from fully anonymized donors with informed consent. PBMCs were flushed from the leukocyte cones using a syringe and isolated using density gradient centrifugation.

### *In vitro* generation of macrophages

Murine BMDMs: BM cells were flushed from the femurs and tibias with cold PBS, filtered through a 45-μm strainer, pelleted by centrifugation, and resuspended in complete DMEM supplemented with 20 ng/ml of M-CSF (BioLegend). Medium was changed on day 4 of culture, and cells were harvested on day 6.

Human MDMs: CD14^+^ cells were enriched from PBMCs by positive selection with CD14 MicroBeads (Miltenyi Biotec) and resuspended in complete RPMI GlutaMAX media (Gibco; 10% FCS, 1 mM sodium pyruvate, 15 mM HEPES, 1% non-essential amino acids, 100 U/ml penicillin, 100 μg/ml streptomycin) supplemented with 50 ng/ml of M-CSF (BioLegend). Medium was changed on days 4 and 6 of culture, and cells were harvested on day 7.

### *In vitro* stimulation and pharmacological modulation of BMDMs, MDMs, and EO771 cells

For *in vitro* experiments with murine cells, 1 × 10^5^ BMDMs were plated in complete media in 96-well plates before polarization with 20 ng/ml IFN-γ (BioLegend), 2 ng/ml LPS (InvivoGen), or 20 ng/ml IL-4 (BioLegend). Where stated, cells were cultured overnight with 2 μg/ml αCD1d (clone 19G11; BioXCell) or rat IgG1 isotype control (clone HRPN; BioXCell).

For *in vitro* experiments with human cells, 5 × 10^4^ MDMs were plated in complete media in 96-well plates. Cells were cultured overnight with 10 μg/ml αCD1d (clone 51.1; BioLegend) or mouse IgG2b isotype control (clone MPC-11; BioLegend) before polarization with 50 ng/ml IFN-γ (BioLegend) or 20 ng/ml IL-4 (BioLegend).

For proliferation analysis, 1 × 10^5^ EO771 tumor cells were plated in complete media in 24-well plates prior to incubation with αCD1d or isotype control, at indicated concentrations and time points prior to flow cytometry.

### *In vivo* tumor cell implantation and therapeutic intervention

EO771 or 4T1 cells were injected into the right fourth inguinal mammary fat pad of female mice at 4 × 10^5^ cells in 50 μl Cultrex Basement Membrane Extract (Bio-Techne). Tumor growth was monitored at regular intervals by measuring the length and width of the tumor using digital calipers. Tumor volume was calculated using the following formula: Volume = (Width^2^ × Length)/2. At the indicated endpoints (days 10−15), mice were euthanized and tumor, spleen, and inguinal lymph nodes were harvested and weighed prior to further processing.

For *in vivo* antibody-mediated blocking experiments, mice were randomized and i.p. injected with the following antibodies (200 μg/mouse/dose, BioXCell): αCD1d (clone 19G11), αPD-1 (clone RPM1-14), αIFN-γ (XMG1.2), αIFNAR (MAR1-5A3), or the respective isotype controls. Mice were injected every 3–4 days as indicated in the figures.

### Tumor processing and flow cytometry

For flow cytometry–based characterization of immune infiltrates, tumors were mechanically dissociated and enzymatically digested into single-cell suspensions using Mouse Tumor Dissociation Kit (Miltenyi Biotec), followed by positive selection of leukocytes using CD45 MicroBeads (Miltenyi Biotec), according to the manufacturer’s protocols. Alive—positively selected—cells were counted in a hemocytometer using trypan blue. The total number of CD45^+^ cells was related to the tumor volume. For detection of intracellular cytokines, 1 × 10^6^ cells were incubated with brefeldin A (Sigma-Aldrich) for 4 h at 37°C in 5% CO_2_ prior to flow cytometry staining.

Fc-block was performed with anti-CD16/32 (TruStain FcX, BioLegend), and dead cells were excluded from the analyses using Zombie fixable viability dye (BioLegend). Antibody staining was performed in FACS buffer (1% FBS, 1% BSA, 0.02% sodium azide, PBS) using the following antibodies from BioLegend unless specified otherwise (at a dilution of 1:200; clones are indicated in brackets): anti-mouse antibodies: B220 (RA3-6B2), CD1d (1B1), CD11b (M1/70), CD11c (N418), CD45.1 (A20), CD45.2 (104), CD24 (M1/69), MHCII (I-A/I-E, M5/114.15.2), Sca-1 (E13-161.7), Ly-6C (HK1.4), Ly-6G (1A8), CD64 (X54-5/7.1), F4/80 (BM8), CD206 (C068C2), NK1.1 (PK136), TCRβ (H57-597), TCRγδ (GL3), IFN-γ (XMG1.2), granzyme B (QA16A02), CD19 (1D3/CD19), CD3 (145-2C11), CD4 (GK1.5), CD8 (53–6.7), CD127 (A7R34), Foxp3 (FJK-16s, eBioscience). CD1d tetramers (loaded with PBS-57; CD1d-tet-PBS-57) were provided by the National Institutes of Health Tetramer Facility. For intracellular cytokine and Foxp3 staining, cells were fixed and permeabilized using BD Cytofix/Cytoperm Solution Kit (BD Biosciences) and Foxp3/Transcription Factor Staining Buffer Set (eBioscience), respectively, according to the manufacturer’s instructions. For proliferation assessment, cells were labeled with CellTrace Violet Cell Proliferation Kit (Invitrogen) according to the manufacturer’s protocol. Flow cytometry data were collected on a BD Fortessa cytometer (BD Biosciences) and were analyzed with FlowJo software (TreeStar).

### qPCR

RNA extraction was performed using RNeasy Mini Kit (Qiagen). cDNA was synthesized with iScript Select cDNA Synthesis Kit (Bio-Rad), and gene expression was determined with iTaq Universal SyBR Green Supermix (Bio-Rad) and the primers included in Table S1 (m, mouse; h, human). Reactions were run in a real-time PCR system (ABI7900HT; Applied Biosystems).

### GSEAs

Bulk RNA-seq data from WT and CD1d-KO pMacs were published previously (Brailey et al., 2022) in Gene Expression Omnibus (GEO) under the accession number GSE215837. GSEAs on a preranked list of genes were performed using the Broad Institute GSEA tool (https://www.gsea-msigdb.org/gsea/index.jsp).

### Myeloid cell sorting and scRNA-seq

Tumors were collected from three WT and three CD1d-KO mice at day 15 after orthotopic injection. CD45^+^ immune infiltrates were isolated, and myeloid cells were sorted as CD11b^+^ and/or CD11c^+^CD45^+^NK1.1^−^CD19^−^CD3^−^Ly6G^−^Siglec-F^−^Zombie^−^ using a BD FACSAria II (BD Biosciences). For all samples, cell viability exceeded 80% prior to loading. Single-cell suspensions were processed using the Chromium Next GEM Single Cell 3′ Reagent Kit version 3.1 (Dual Index) and loaded onto a Chromium Chip G (10x Genomics), according to the manufacturer’s instructions. A target of 10,000 cells per sample was loaded per channel. Libraries were prepared using the 10x Chromium platform and sequenced with 150-bp paired-end reads on the NovaSeq X Plus system (Illumina).

Sequencing data associated with this paper have been deposited in the National Center for Biotechnology Information’s GEO repository under the accession number GSE317860.

### Analysis of scRNA-seq data

The raw FASTQ files were processed using Cell Ranger version 9.0.1, where each sample was mapped to mouse reference GRCm39 (2024-A). All downstream analyses were performed in R version 4.5.1 except for trajectory analyses (see below). Cell Ranger–filtered feature–barcode matrices were loaded into Seurat (version 5.3.1). Cells with fewer than 200 or more than 7,500 detected genes or >5% mitochondrial content were excluded. Raw counts were normalized using SCTransform, and cells were clustered using the Louvain algorithm after principal component analysis of 2,000 variable features. UMAP was used for visualization (McInnes et al., 2020, *Preprint*). Marker genes were identified using FindAllMarkers() (Wilcoxon rank-sum test). Myeloid clusters were subsetted and reprocessed. Cell-type proportions were calculated per condition and displayed as stacked bar plots. Expression scores for inflammatory (Duong et al., 2022; Elewaut et al., 2025; Jablonski et al., 2015) and immunosuppressive TAM signatures (Jablonski et al., 2015; van Elsas et al., 2024; Zhang et al., 2020) were computed using AddModuleScore() and visualized on UMAP.

RNA velocity matrices for trajectory analyses were generated using velocyto with BAM files generated during Cell Ranger processing. Spliced/unspliced transcript counts were then merged with the single-cell object (Seurat object converted to AnnData in scanpy). RNA velocities of WT and CD1d-KO were computed, separately, using dynamo version 1.4.3.

For pseudobulk differential expression, single-cell counts were aggregated per sample using Seurat’s AggregateExpression function and analyzed with DESeq2 (version 1.48.2). GSEA was performed using fgsea (version 1.34.2, MSigDB) and GSEA (version 4.4.0, Broad Institute, 1,000 permutations). Upstream regulator analysis was performed using Ingenuity Pathway Analysis.

The murine inflammatory monocyte signature (c3_Inflam-Mono signature) comprised the top 50 DEGs in the inflammatory monocyte cluster (c3). The DEG signature (DEG-c3_Inflam-Mono signature) comprised all significantly upregulated genes (*n* = 71) in CD1d-KO versus WT inflammatory monocytes (c3). Mouse genes were converted to human orthologs using BioMart (Ensembl December 2021). Human scRNA-seq data from Wu et al. (2021) (Single Cell Portal) with provided cell-type annotations were processed in Seurat. Myeloid cells were scored for c3_Inflam-Mono signature using ssGSEA (GSVA version 2.2.1). The human c9_CXCL10-Mac signature was defined from the top 100 DEGs from Myeloid_c9_macrophage_2_CXCL10 population from Wu et al. (2021).

METABRIC data (cBioPortal, EGAS00000000083) were log-transformed and scored for mouse c3_Inflam-Mono, mouse DEG-c3_Inflam-Mono, and human c9_CXCL10-Mac using GSVA with z-score normalization. TNBC patients were stratified by quartiles. Kaplan–Meier curves with log-rank tests compared overall and relapse-free survival. Hazard ratios and 95% confidence intervals were estimated using univariable Cox proportional hazards regression. Multivariate Cox regression was adjusted for age, tumor grade, stage, size, chemotherapy, and radiotherapy. Analysis was restricted to grade 2–3, stage 1–2 patients (*n* = 98–102, events = 35–55 depending on outcome and signature).

The Bassez et al. breast cancer immunotherapy dataset (Bassez et al., 2021, EGAS00001004809) was processed in Seurat using LogNormalize with 2,000 variable features. After principal component analysis and clustering, cells were visualized by time point and response status. In this dataset, T cell clonotype expansion was used as a surrogate for treatment response as described in the original manuscript. Cluster 0 frequencies were compared between responders and nonresponders using Mann–Whitney U tests. Pseudobulk profiles were generated per patient for each time point (pre- and on-treatment). Pseudobulk data were normalized to counts per million and log2-transformed. Mouse c3_Inflam-Mono and human c9_CXCL10-Mac signature scores were calculated for all cells using ssGSEA. Cluster 0 was subsetted, and mouse DEG-c3_Inflam-Mono signature scores were calculated using ssGSEA. Signature scores were compared between responders and nonresponders using Mann–Whitney U tests for each time point (pre- and on-treatment).

### Statistical analysis

Statistical tests were performed using GraphPad Prism Software. Comparison between groups was performed using two-tailed paired or unpaired Student’s *t* tests, or one-way or two-way ANOVA tests as appropriate unless otherwise stated. Values of P < 0.05 were considered statistically significant. For survival analyses, a log-rank Mantel–Cox test and for response rates Fisher’s exact test were performed.

### Online supplemental material

Fig. S1 shows data related to Fig. 1, including gaiting strategy for myeloid and lymphoid populations in EO771 tumors. Fig. S2 shows data related to Fig. 2 including CD1d expression in human and murine cells in breast cancer and quantification of immune infiltrates after αCD1d administration to tumor-bearing mice. Fig. S3 shows data related to scRNA-seq analyses from murine cells (related to Fig. 3), gene expression in inflammatory monocytes (related to Fig. 4), and analyses of myeloid cell populations in human breast cancer (related to Fig. 5). Table S1 shows primer sequences.

## Supplementary Material

Table S1 shows primer sequences.

## Data Availability

Sequencing data have been deposited in the National Center for Biotechnology Information’s GEO repository under the accession number GSE317860. All other data are available in the article itself and its supplementary materials.
