# Supplementary material for: CD1d remodels the tumor-infiltrating myeloid populations controlling antitumor immunity
Source: J Exp Med. 2026 Aug 13;223(9):e20260216. doi: 10.1084/jem.20260216 (PMC13521511; doi:10.1084/jem.20260216)
Supplement: Supplementary file 1 [file jem_20260216_tables1.docx]

Table S1. Primer sequences

| **Target** | **Forward Sequence** | **Reverse Sequence** |
| --- | --- | --- |
| *mHprt* | TCAGTCAACGGGGGACATAAA | GGGGCTGTACTGCTTAACCAG |
| *mNos2* | CCACCCGAGCTCCTGGAAC | CCCTCCTGATCTTGTGTTGGA |
| *mArg1* | CAGATATGCAGGGAGTCACC | CAGAAGAATGGAAGAGTCAG |
| *mChi3l3* | TTTGTCCTTAGGAGGGCTTCCTC | TCACAGGTCTGGCAATTCTTCTG |
| *mCxcl10* | CCAAGTGCTGCCGTCATTTTC | GGCTCGCAGGGATGATTTCAA |
| *mIl12* | AACTTGAGGGAGAAGTAGGAATGG | GGAAGCACGGCAGCAGAATA |
| *hBACTIN* | CATGTACGTTGCTATCCAGGC | CTCCTTAATGTCACGCACGAT |
| *hCD80* | CTGCCTGACCTACTGCTTTG | GGCGTACACTTTCCCTTCTC |
| *hTGM2* | CCTCGTGGAGCCAGTTATCAA | GTCTGGGATCTCCACCGTCTTC |
